# Successful systemic thrombolytic therapy for massive pulmonary embolism in a patient with breast cancer, brain metastasis, and thrombocytopenia: A case report

**DOI:** 10.1002/ccr3.1629

**Published:** 2018-06-05

**Authors:** Toktam Alirezaei, Behzad Hajimoradi, Mehdi Pishgahi, Seyyed Mojtaba Nekooghadam, Mohamad Golmohamadi

**Affiliations:** ^1^ Cardiology Department of Shohaday‐e‐Tajrish Hospital Shahid Beheshti University of Medical Sciences Tehran Iran; ^2^ Internal Department of Shohaday‐e‐Tajrish Hospital Shahid Beheshti University of Medical Sciences Tehran Iran

**Keywords:** brain metastasis, massive pulmonary embolism, systemic thrombolysis, thrombocytopenia

## Abstract

A female with massive PTE and absolute contraindication for thrombolytic did not meet guidelines due to unavailability of catheter or surgical embolectomy and giving thrombolytic as a last resort to save a life. In such cases, physicians should consider to act outside of the guidelines to save a life.

## 1 INTRODUCTION

Venous thromboembolism (VTE) is a common complication of cancer and is a major cause of morbidity and one of the leading causes of mortality in terminal cancer patients.

Treatment of thromboembolic disease especially life‐threatening conditions such as massive pulmonary embolism in cancer patients is challenging.

It is common for clinicians to be faced with the dilemma of how to manage cancer patients with massive pulmonary embolism in life‐threatening settings. In the present case, the problem was further complicated by brain metastasis and thrombocytopenia both of which have a dramatic impact in the risk of intracranial hemorrhage after thrombolysis.

## 2 CLINICAL CASE REPORT

A 65‐year‐old female, known case of metastatic breast cancer with brain involvement, presented to our emergency department because of dyspnea, tachypnea, tachycardia and hypotension since 2 hours ago.

She had history of triple positive (ER+, PR+, HER2+) breast cancer and left radical mastectomy 15 years ago after which systemic chemotherapy and hormone therapy (Letrozole/Tamoxifen) was implemented. Three years before current presentation, bilateral hystero‐salpingo‐ovoforectomy, was performed. About 2 months ago, routine clinical examination by her oncologists revealed left axillary lymphadenopathy (2 × 3 cm) with fixed, firm and nontender node and an additional fixed mass (1 × 3 cm) in the anterior left forearm. Both lesions were excisionally biopsied. Further Imaging showed bilateral hilar lymphadenopathy and multiple metastatic lesions in the liver & abdomen and chest. PET scan showed 2 metastatic lesions in brain.

Pathologic examination of excised node revealed metastatic breast cancer (ER+/HER2+) and salvage chemotherapy with Paclitaxel 100 mg and Gemcitabin 1000 mg weekly plus Herceptin (Trastuzumab) 440 mg and Perjecta (Pertuzumab) 14 mg/kg tri weekly was initiated. One day after the first dose of Herceptin and Perjecta and the fourth dose of Gemcitabin and Paclitaxel, the right lower extremity swelling developed and subsequent Compression ultrasound and Doppler studies revealed acute extensive thrombus formation in the superficial femoral vein of the right leg. The patient was admitted and anticoagulation therapy with LMVH (60 mg BID) was initiated under the supervision of an oncologist at a private hospital. Preliminary laboratory tests revealed mild pancytopenia with a platelet count of 60 000 that was attributed to recent chemotherapy.

Two days after anticoagulation initiation she was discharged home.

Shortly after discharge, she presented to our emergency department with complaint of severe shortness of breath beginning abruptly 2 hours ago.

Her vital signs upon arrival to the emergency department showed a systolic blood pressure of 70 mm Hg, a heart rate of 155 beats per minute, a respiratory rate of 40 per minute & oxygen saturation of 70% in ambient air. She was apparently pale, agitated, diaphoretic and unable to speak in full sentence. Jugular veins were distended and cardiac exam showed moderate tachycardia and a right ventricular heave.

Pulmonary examination was unremarkable except for decreased breath sounds at the mid‐zone of the left lung and the base of right lung.

**Figure 1:**
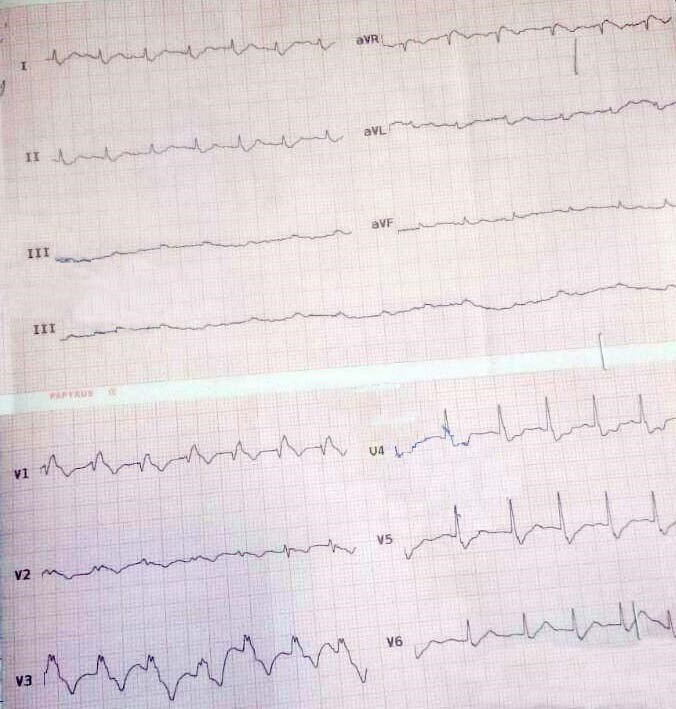
ECG shows sinus tachycardia and right bundle branch block

Radial pulses were very weak bilaterally with no palpable pulse in lower extremities. The initial electrocardiogram showed sinus tachycardia at a rate of 135 per minute, right bundle branch block with a QRS duration of 130 ms and right axis deviation of 110 degrees (Figure 1).

Bedside echocardiography performed despite severe agitation of the patient demonstrated preserved EF (EF = 50%‐55%), severe right ventricular (RV) dilation and estimated pulmonary artery pressure (PAP) of 45‐50 mm Hg.

Repeat laboratory findings showed Hemoglobin: 8 (g/dL), Platelets count: 50/mm^3^.

Immediately a 500 cc bolus of normal saline was administered and norepinephrine was initiated as vasopressor of choice.

**Figure 2:**
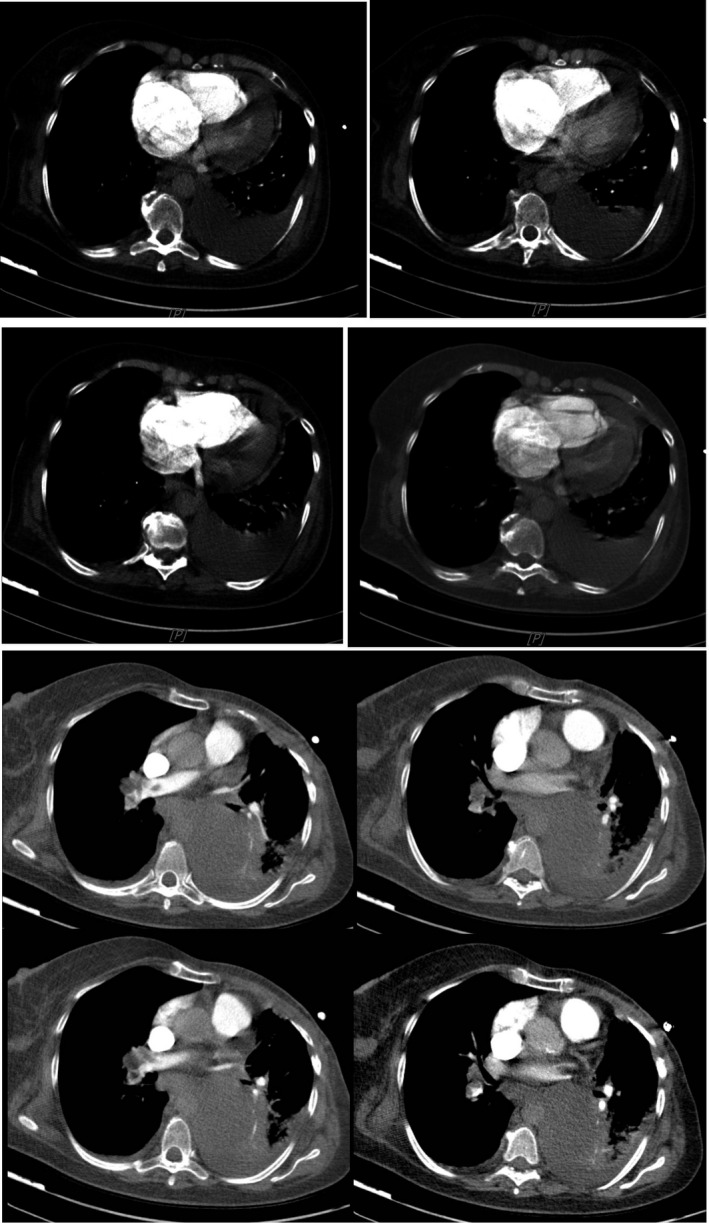
Pulmonary CT angiography demonstrates bilateral pulmonary thromboemboli with RV strain

Pulmonary CT angiography (CTA) confirmed large bilateral pulmonary thromboemboli with radiological evidence of RV strain (Figure 2).

According to hemodynamic compromise resulting from the massive pulmonary embolism and absolute contraindication for thrombolytic therapy due to brain metastases and concurrent thrombocytopenia, she became candidate for emergent catheter or surgical embolectomy. Surgical consult with cardiothoracic surgeon was requested but due to critical condition of the patient complicated by refractory hypoxia and unstable hemodynamic parameters surgery and anesthesiology team declared the patient as inoperable and there was no equipment and specialist for catheter embolectomy in our center.

Ultimately the critical decision was made, despite absolute contraindications, to treat the patient with systemic thrombolysis. Alteplase was selected as thrombolytic of choice and was prescribed according to approved dosage for massive PTE (100 mg infusion over a 2 hours’ period).

**Figure 3:**
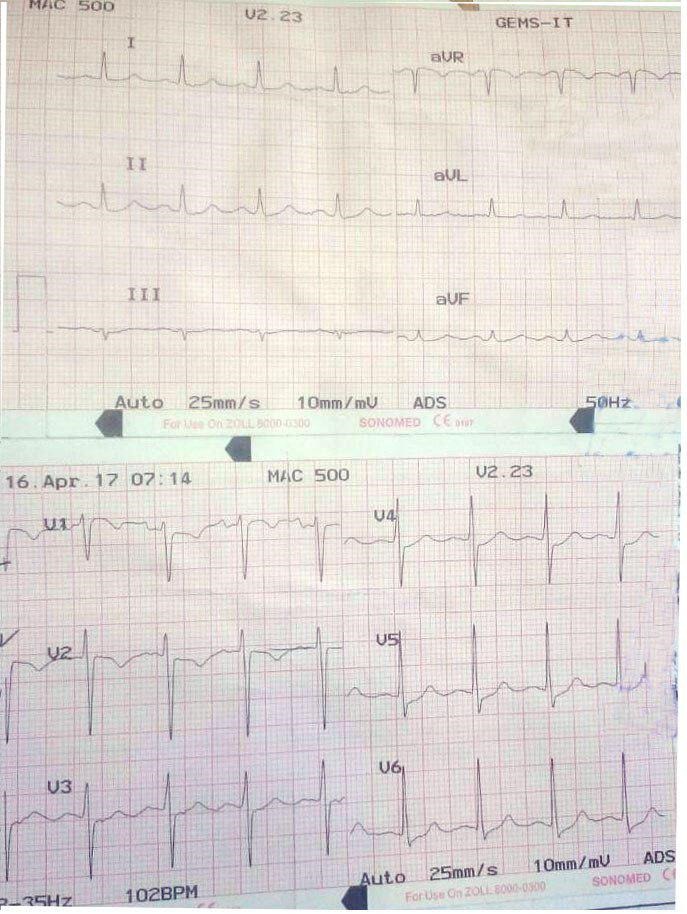
ECG shows normal sinus tachycardia

Approximately 1 hour after termination of thrombolytic therapy, the norepinephrine was discontinued. Blood pressure returned to 110/70 mm Hg, with a heart rate of 100 beats per minute and a respiratory rate of 20 per minute with an oxygen saturation of 90% in ambient air. Electrocardiogram now shows sinus tachycardia at a rate of 100 per minute, normal QRS duration and normal axis, Figure 3.

Heparin infusion was initiated with half‐dose because of concurrent thrombocytopenia.

After 30 hours, heparin infusion stopped due to a drop in platelet count (platelet count: 23 000/mm^3^), consulted with hematologist colleagues, they believed it was due to recent chemotherapy, so 10 units of platelets were transfused. One day later platelet count reached 123 000/mm^3^, heparin infusion was resumed, and after 24 hours it changed to a therapeutic dose of subcutaneous enoxaparin (LMWH with a dose of 60 mg twice a day). The patient continued to improve clinically and was discharged from hospital after 5 days with LMWH. Last echocardiography performed immediately before discharge demonstrated mild RV enlargement & dysfunction and pulmonary artery pressure of 20‐25 mm Hg. One week after discharge, laboratory findings showed Hemoglobin: 11.5 (g/dL), Platelet count: 227/mm.

## 3 DISCUSSION

Venous thromboembolism (pulmonary embolism and deep venous thrombosis) is a common complication of various cancers and their treatments.^1^, ^2^ Malignancies are associated with a 4‐fold increase in VTE incidence^3^ and VTE affects up to 20% of cancer patients, such that it is one of the major causes of morbidity and mortality in these population.^4^

Metastatic disease at the time of diagnosis is considered to be the strongest predictor of VTE within the first year of diagnosis and is associated with a 1.4‐21.5‐fold higher risk of VTE according to the type of cancer.^5^, ^6^, ^7^, ^8^, ^9^, ^10^

Cancer‐related VTE is associated with higher rates of mortality or morbidity, in part due to increased bleeding complications imposed by anticoagulation/fibrinolytic therapy compared with VTE in patients without cancer^4^, ^11^ it is also a marker of more advanced disease and portends a poor prognosis.^12^

Metastatic brain lesions are more prone to spontaneous intracranial hemorrhage compared with primary brain tumors.^13^ However, the risk of intracranial hemorrhage from breast cancers brain metastases is relatively low (1%‐5%).^14^, ^15^

In recent decades, owing to striking developments in the field of treatment of metastatic breast cancer, long‐term survival can be achieved in many patients.^16^ Furthermore, the increased prevalence of brain metastases is becoming a major impediment to improve quality of life for many breast cancer patients.

International society guidelines addressing management of pulmonary embolism, generally propose 2 viable options in the management of massive PTE: (i) thrombolysis, (ii) surgical embolectomy. Systemic thrombolysis leads to rapid resolution of thrombi and improves hemodynamic instability.^17^, ^18^ Despite this recommendations, in a study of 108 patients with massive pulmonary embolism, 2‐thirds of the patients did not receive thrombolysis or embolectomy.^19^

Improvement in the survival of cancer patients may lead to an increase in malignancy‐associated VTE episodes, many of which are complicated by concomitant metastatic involvement of brain. In conditions such as massive pulmonary embolism, the patients need emergency treatments. Systemic thrombolysis has absolute contraindication in the cases of brain metastases and emergency embolectomy is not available in all hospitals ‐and if available‐ many surgeons and anesthesiologists are reluctant to accept the risk of surgery. Act outside of the guidelines to save a life, Therefore, in life‐threatening conditions, clinicians may be faced with the question of how to manage patient with massive pulmonary embolism and they have to act outside of the guidelines to lifesaving.^20^

In the current case, we encountered a management crisis in a patient with massive pulmonary embolism who was at extremely high risk for bleeding complications of thrombolytic therapy and at the same time, no other acceptable recourse was available. Ultimately the patient survived the jeopardy of this double‐edge remedy. By this case report, we reported successful outcome of thrombolytic therapy as a last resort in massive PTE despite its contraindication.

## CONFLICT OF INTEREST

The authors have no conflict of interests to declare.

## AUTHORSHIP

TA: analyzed data, drafted, did background research, and revised the manuscript. BH and MP: involved in patient management. SMN: reviewed results and revised the manuscript. MG: collected history.
